# Complete genome sequencing and assembly of two *Novosphingobium* spp. isolated from the phyllosphere of *Actinidia chinensis* (kiwifruit)

**DOI:** 10.1128/mra.00257-26

**Published:** 2026-07-17

**Authors:** Polina Idelchik, Monica C. Summers, Monica L. Gerth

**Affiliations:** 1 School of Biological Sciences, Victoria University of Wellington 8491, Wellington, New Zealand; University of Strathclyde, Glasgow, United Kingdom

**Keywords:** kiwifruit, phyllosphere microbiome, *Novosphingobium*, long-read sequencing, *Actinidia*

## Abstract

Two *Novosphingobium* strains designated as TK46 and TK71 were isolated from kiwifruit leaves (*Actinidia chinensis* var. *chinensis* ‘Zesy002,’ commonly known as Gold3) in Te Kaha, New Zealand. We report their complete genome sequences, providing valuable resources for understanding their ecology and potential applications in plant-associated microbial research.

## ANNOUNCEMENT

*Novosphingobium* is a genus of aerobic, rod-shaped, gram-negative bacteria with versatile metabolic and plant growth promotion capabilities (1–3). As part of an investigation into the epiphytic phyllosphere microbiome of kiwifruit (*Actinidia chinensis*), we isolated two *Novosphingobium* strains designated as TK46 and TK71.

Strains were collected in April 2023 from leaves of an *Actinidia chinensis* var. *chinensis* ‘Zesy002’ vine in a commercial orchard in Te Kaha, New Zealand (37.736175°S, 177.696475°E). Primary isolation was performed in the field by pressing individual leaves onto Luria-Bertani (LB) agar (1.5% w/v) supplemented with cycloheximide (0.177 mM). Gloves and sampling tools were disinfected with bleach between samples. Agar plates were transported to the laboratory at ambient temperature within 24 h of collection, after which all cultivation steps were performed at 22°C.

Following 3 days of incubation, individual colonies of distinct morphotypes were selected. The reported isolates were derived from separate leaves. To ensure clonal purity, a single colony of each morphotype was subjected to one round of restreaking on LB agar (1.5% w/v). For cryopreservation, single colonies were inoculated into LB broth, cultured overnight, mixed 1:1 with sterile 50% (v/v) glycerol, and stored at −70°C.

After a few months, the cryopreserved isolates were revived on LB agar (1.5% w/v). A single colony from each isolate was inoculated into LB broth and incubated overnight with shaking at 200 rpm. Genomic DNA was extracted using the Wizard Genomic DNA Purification Kit (Promega) according to the protocol for gram-negative bacteria.

Sequencing libraries were prepared according to the Oxford Nanopore Technologies (ONT) “Ligation Sequencing gDNA-Native Barcoding (SQK-NBD114.24)” protocol; no prior size selection or fragmentation was performed. Sequencing was performed on a MinION Mk1B instrument using an R10.4.1 flow cell.

The basecaller Dorado (v0.9.5, model dna_r10.4.1_e8.2_400bps_sup@v5.0.0) (4) with the duplex option was used for basecalling; reads with a Q-score below 10 were discarded. No other quality control measures were applied. The basecalled ONT reads were assembled with Flye (v2.9.5) (5), and the assembly was polished once with Medaka (v2.0.1) (6) using the same reads. Replicon circulation and overlap trimming were performed as part of the assembler’s default workflow. Genome rotation was not performed. Default parameters were used for all software. No down-sampling or other reduction methods were used during assembly.

The genomes were annotated using the National Center for Biotechnology Information (NCBI) Prokaryotic Genome Annotation Pipeline (PGAP, v6.10) (7). Assembly of the TK46 genome yielded two circular replicons, a 3.81 Mb chromosome and a 301.74 kb plasmid. Assembly of the TK71 genome yielded three circular replicons: a chromosome with a size of 3.44 Mb, a plasmid with a size of 134.7 kb, and a putative chromid of size 2.34 Mb. The putative chromid was identified by its size and the presence of ribosomal genes (8, 9). The presence of plasmids was confirmed with Plasmer (v0.1) (10).

Assembly quality was estimated using BUSCO (v5.8.2) (11) with *Novosphingobium*_odb12 lineage and CheckM (v1.2.4) (12) with the marker lineage *Sphingomonadales* (UID3310). Average nucleotide identity (ANI) was calculated using the NCBI ANI workflow for prokaryotic genome assemblies (13). Sequencing, assembly, and annotation statistics are presented in Table 1.

**TABLE 1:** Genome sequencing and assembly statistics

Parameter	*Novosphingobium* sp. TK46	*Novosphingobium* sp. TK71
Sequencing statistics		
Total number of reads	94,413	97,175
Sequencing *N*_50_ (bp)	19,001	17,858
Assembly description		
Average genome coverage	195×	136×
Number of contigs	2	3
Assembly quality		
Assembly *N*_50_ (bp)	3,807,432	3,436,332
BUSCO (completeness)	98.1	99.8
CheckM completeness^*a*^	96.21%	96.38%
CheckM contamination^*a*^	6.89%	10.13%
Taxonomy		
Average nucleotide identity (ANI) (%)	82.55%	85.31%
Best match type-strain	*Novosphingobium fluoreni*	*Novosphingobium lindaniclasticum*
Best match assembly	GCA_014196615.1	GCA_000445125.1
Genome description		
Genome size (bp)	4,109,176	5,915,877
G + C content (%)	64	64.5
Total no. of genes	3,926	5,345
Total no. of coding sequences	3,862	5,262
No. of proteins	3,818	5,211
No. of pseudogenes	44	51
Total no. of RNAs	64	83
No. of rRNAs	9	15
No. of tRNAs	52	65
No. of non-coding RNAs	3	3

^*a*^ Based on *Novosphingobium* CheckM (v1.2.4) (12) marker set.

## Data Availability

All data are available under NCBI BioProject PRJNA1337265. TK46: sampling metadata (BioSample SAMN52585905), raw reads (SRR35847487), genome assembly files (JBSZKB000000000.1; first version described here). TK71: sampling metadata (BioSample SAMN52585906), raw reads (SRR35847486), genome assembly files (JBSZKA000000000.1; first version described here).
